# Curcumol attenuates liver sinusoidal endothelial cell angiogenesis via regulating Glis‐PROX1‐HIF‐1α in liver fibrosis

**DOI:** 10.1111/cpr.12762

**Published:** 2020-03-02

**Authors:** Xiang Yang, Zhimin Wang, Jun Kai, Feixia Wang, Yan Jia, Shijun Wang, Shanzhong Tan, Xikun Shen, Anping Chen, Jiangjuan Shao, Feng Zhang, Zili Zhang, Shizhong Zheng

**Affiliations:** ^1^ Jiangsu Key Laboratory for Pharmacology and Safety Evaluation of Chinese Materia Medica School of Pharmacy Nanjing University of Chinese Medicine Nanjing China; ^2^ Shandong University of Traditional Chinese Medicine Jinan China; ^3^ Nanjing Hospital Affiliated to Nanjing University of Chinese Medicine Nanjing China; ^4^ Suzhou Hospital of Traditional Chinese Medicine Suzhou China; ^5^ Department of Pathology School of Medicine Saint Louis University St. Louis MO USA; ^6^ Jiangsu Key Laboratory of Therapeutic Material of Chinese Medicine School of Pharmacy Nanjing University of Chinese Medicine Nanjing China

**Keywords:** angiogenesis, curcumol, hedgehog, HIF‐1α, liver sinusoid endothelial cells, PROX1

## Abstract

**Objective:**

Hepatic sinusoidal angiogenesis owing to dysfunctional liver sinusoidal endothelial cells (LSECs) accompanied by an abnormal angioarchitecture is a symbol related to liver fibrogenesis, which indicates a potential target for therapeutic interventions. However, there are few researches connecting angiogenesis with liver fibrosis, and the deeper mechanism remains to be explored.

**Materials and Methods:**

Cell angiogenesis and angiogenic protein were examined in primary LSECs of rats, and multifarious cellular and molecular assays revealed the efficiency of curcumol intervention in fibrotic mice.

**Results:**

We found that curcumol inhibited angiogenic properties through regulating their upstream mediator hypoxia‐inducible factor‐1α (HIF‐1α). The transcription activation of HIF‐1α was regulated by hedgehog signalling on the one hand, and the protein stabilization of HIF‐1α was under the control of Prospero‐related homeobox 1 (PROX1) on the other. A deubiquitinase called USP19 could be recruited by PROX1 and involved in ubiquitin‐dependent degradation of HIF‐1α. Furthermore, our researches revealed that hedgehog signalling participated in the activation of PROX1 transcription probably in vitro. Besides, curcumol was found to ameliorate liver fibrosis and sinusoid angiogenesis via hedgehog pathway in carbon tetrachloride (CCl_4_) induced liver fibrotic mice. The protein expression of key regulatory factors, PROX1 and HIF‐1α, was consistent with the Smo, the marker protein of Hh signalling pathway.

**Conclusions:**

In this article, we evidenced that curcumol controlling LSEC‐mediated angiogenesis could be a promising therapeutic approach for liver fibrosis.

## 1 INTRODUCTION

Angiogenesis is a hypoxia‐induced and growth factor–dependent process in which endothelial cells are budded, accompanied by migration, expansion and lumen formation from the original vascular structure. Liver fibrosis is a common consequence of injury and repair reaction of various chronic liver diseases (CLDs), and is related to angiogenesis closely.^1^ There are a large number of pseudolobular fibrous nodules in hepatic tissue during liver fibrosis, which lead to hepatic sinusoidal blood flow disorder and oxygen delivery reduction. These changes make liver intrinsic cells, such as hepatocytes and activated hepatic stellate cells (HSCs), secret angiogenic factors in order to regulate endothelial angiogenesis. Of note, neovascularization destroys hepatic architecture and promotes sinusoidal remodelling, aggravating liver fibrosis. As a result, pathological angiogenesis and fibrogenesis develop in parallel during progression of CLD.^2^ Therefore, the inhibition of pathological angiogenesis has also become a significant method to alleviate liver fibrosis.

The LSECs account for the vast majority of liver non‐parenchymal cells and are directly involved in hepatic angiogenesis.^3^ The natural LSECs lack an organized basement membrane and have numerous fenestrae grouped into sieve plates, which benefit to mass exchange of hepatic sinusoid and resist exogenous invasion. During the fibrogenic progression of CLD, different factors have made LSEC lose their distinctive morphology through a process named capillarization. Neovascularization exacerbates liver hypoxia and resistance to blood flow, which increases transcription of many hypoxia‐sensitive pro‐angiogenesis genes such as vascular endothelial growth factor (VEGF),^4^ platelet‐derived growth factor (PDGF) and angiopoietin.^5^ All these processes are regulated by the hypoxia‐inducible factor‐1α (HIF‐1α).^6^ Basic and clinical research also showed that inhibition of LSEC angiogenesis and HSC paracrine effects could alleviate liver fibrosis.^5^, ^7^

The hedgehog (Hh) pathway is a conserved morphogenic signalling pathway that modulates the fate of LSECs, including capillarization and angiogenesis.^8^, ^9^ There is growing evidence showed that liver injury can activate Hh pathway tremendously.^10^ The interaction between increasing sonic hedgehog protein (SHH) and membrane receptor Patched can replace the combination of Patched between intracellular co‐receptor–like molecule Smoothened (Smo). The latter eventually results in the initiation of Glis‐dependent canonical hedgehog signalling.^11^ In this progress, Gli‐family transcription factors (Gli1, Gli2 and Gli3) that translocate to nucleus from cytoplasm, which regulate the Hh target gene transcription, affect cell viability, proliferation and differentiation. Previous studies disclosed that Hh can activate pro‐angiogenic gene via regulating HIF‐1α to promote HSC angiogenesis.^12^ The potential mechanisms of Hh pathway regulating the phenotype of LSEC, however, remain uncovered. Here, we have mainly investigated how the Hh pathway regulates LSEC angiogenesis.

Curcumol is extracted from the roots of the herb called Rhizoma Curcumae, and it possesses a variety of pharmacological activities including anti‐inflammatory and anti‐tumour effect. Previous researches had revealed that curcumol can inhibit the proliferation of nasopharyngeal carcinoma CNE‐2 cells by regulating insulin‐like growth factor 1 receptor (IGF‐1R) and its downstream PI3K/Akt/GSK‐3beta pathway.^13^ Besides, curcumol can suppress breast cancer cell metastasis by inhibiting the expression of MMP‐9.^14^ These studies suggested that curcumol is possible to participate in cell proliferation and migration correlated with angiogenesis closely. Moreover, a recent study demonstrated that curcumol is capable of alleviating liver fibrosis via triggering HSC necroptosis.^15^ Given the anti‐fibrotic effect of curcumol, we have investigated whether curcumol can inhibit hepatic fibrosis via regulating LSEC angiogenesis and elucidated the underlying mechanisms.

## 2 MATERIALS AND METHODS

### 2.1 Cell culture

Primary LSECs were isolated from male SD rats as described.^8^ Isolated LSECs were cultured in complete medium made from Dulbecco's modified Eagle's medium (DMEM) (Procell) supplemented with foetal bovine serum (FBS) (Gibco) (9:1) and 1% double antibiotics (Streptomycin and aspirin) and placed in 37°C and 5% CO_2_ incubator. LSECs entering logarithmic growth period were used in experiments. Cell morphology was assessed via microscope with a Leica Qwin System (Leica).

### 2.2 Cell viability assay

LSECs were planted in 96‐well plates (about 10^4^ cells per well) and treated with various reagents when they would be 70%‐90% confluent for 24 hour. MTT (Biosharp) was dissolved in PBS at a concentration of 5 mg/mL. The configured MTT was dissolved into the total solution and added to the 96‐well plates (20 μL per well). Then, 96‐well plates were put in incubator for 6 hours. Dimethyl sulphoxide (DMSO) was added to the 96‐well plates (150 μL per well) to dissolve the bottom crystal. The spectrophotometric absorbance was measured by a spectrophotometer (Molecular Devices) at 490 nm. We set up six duplicate wells for each group in this experiment.

### 2.3 Western blot analysis

The LSEC or fresh liver from mice was dissolved with lysis buffer consisting RIPA, PMSF and protease inhibitor (100:1:1). Protein concentration was measured by BCA kit (Beyotime Biotech). We evaluated the level of target protein expression according to the depth of bands until the β‐actin had the same expression. The strips were quantified with Image Lab (NIH). Nuclear proteins and cytoplasm proteins were separated using a Nuclear and Cytoplasmic Extraction Kit (Wanleibio).

### 2.4 Quantitative reverse transcription (PCR)

TRIzol reagent (Sigma) was used to extract total RNA from treated LSECs, and then, the RNA was subject to reverse transcription to cDNA using the kits provided by Yeasen Biotech Co., Ltd after purification. Then, the real‐time PCR was performed using the SYBR Green Master Mix. The glyceraldehyde 3‐phosphate dehydrogenase (GAPDH) was used as the invariant control. The primers of all genes are listed in Table 1.

**Table 1:** Glyceraldehyde 3‐phosphate dehydrogenase (GAPDH) was used as the invariant control. The following primers of all genes are available

Gene	Forward sequence (5′ to 3′)	Reverse sequence (5′ to 3′)
GAPDH	GCATCTTCTTGTGCAGTGCC	TACGGCCAAATCCGTTCACA
HIF‐1α	ACTATGTCGCTTTCTTGG	GTTTCTGCTGCCTTGTAT
PROX1	AGACTTTGACCACCGTGTCC	GCAGGCCTACTATGAGCCAG
Angiopoietin 2	TTGCGACCCCTTCAACTCTG	CCCTTGGGCTTGGCATCACT
PDGF‐B	TGGAGTCGAGTCGGAAAGC	GCACTGCACATTGCGGTTA
VEGFR2	GTCTTATGGCGTTCTGCTC	CCATCCTGTATCCGCTCTT
Patched	GCTCCCAAGCAATACAAA	TCCCAGATGACCGATA
Endothelin‐1	CTGCCACCTGGACATCAT	TTTGGGCTCGGAGTTCTT
Smo	ATGCGTGTTTCTTTGTGGGC	ACACAGGATAGGGTCTCGCT
SHH	GTAACGCTACGAGAGGAGGC	CCTCGCTTCCGCTACAGATT
Hhip	AATGTGAGCCACCTTGTCGT	GGAATGCCCACCGGAAAGAT

### 2.5 Tube formation assay

In tube formation assay, a total volume of 150 μL (10 μg/mL) Corning Matrigel Basement Membrane Matrix covered in 24‐well plates was pre‐cooled for 24 hours, then put in incubator at 37°C for 30 minutes to solidify. LSECs were seeded into wells (nearly 5 × 10^5^ per well) after treated with different reagents. Plates were incubated at 37°C and then observed every hour under microscope, and tube length was measured via ImageJ software (NIH).

### 2.6 siRNA and plasmid transfection

PROX1 siRNA and plasmid were synthesized by Kaiji Biotech (Nanjing, China). PROX1 siRNA (forward: 5′‐CCGGGUUGAGAAUAUCAUUTT‐3′; reverse: 5′‐AAUGAUAUUCUCAACCCGGGC‐3′) or PROX1 plasmid was added in FBS‐free medium for 5 minutes at room temperature. Lipofectamine 2000 reagent (Life Technologies) was mixed with FBS‐free medium at 1:50 for 5 minutes too. Both mixtures were incubated for another 30 minutes. Finally, the LSEC in six‐well plates was covered by transfection mixture for 6 hours in the incubator. Observation of cells carrying fluorescence under the microscope indicates successful transfection. Cells were cultured for another 6 hours after transfection to find its feet.

### 2.7 Co‐immunoprecipitation (Co‐IP) assay

Treated LSECs were extracted at 4°C in RIPA buffer containing protease inhibitors. Cell lysates diluted to 1 mg/mL protein concentration were co‐incubated by IP‐grade antibodies against HIF‐1α (1:200, CST). After gentle shaking at 4°C overnight, protein A/G PLUS‐Agarose (Santa Cruz Biotech) was added to the lysate/antibody mixture and incubated with gentle rocking at 4°C for 4 hours. Then, the immunoprecipitates were collected by centrifugation and washed three times with cell lysis buffer, then boiled for 5 minutes with the same volume of 4× loading buffer. Proteins were resolved by 10% SDS‐PAGE and subjected to Western blotting assays.

### 2.8 Ubiquitination assay

After treated LSECs were incubated for 24 hours, partial groups were treated with MG132 (10 mg/mL) for another 6 hours. Cell lysates diluted to 1 mg/mL protein concentration were pulled down by IP‐grade antibodies against HIF‐1α at 4°C overnight. Proteins were resolved by 10% SDS‐PAGE. We evaluated the ubiquitination levels of treated cells by anti‐ubiquitin antibody.

### 2.9 Animals and procedures

Male ICR mice (20‐25 g body weight) were purchased from Qinglongshan Animal Co, Ltd. All animals were raised in Nanjing University of Chinese Medicine (Nanjing, China) after the experimental procedures received the approval of the institutional and local committee. The whole animals were given humane care according to the National Institutes of Health guidelines. The mice were intraperitoneal injected with a mixture of carbon tetrachloride (CCl_4_) (0.5 mL/100 g body weight) and olive oil [1:9 (v/v)] twice a week to make liver fibrosis model. The lentivirus was caudal vein–injected into partial mice twice in 2 weeks to create Smo protein–overexpressed mice model. Sixty mice were randomly divided into six groups (n = 10), named separately control, CCl_4_, CCl_4_ + vector, CCl_4_ + Smo, CCl_4_ + curcumol and CCl_4_ + Smo+curcumol. The mice in the drug intervention groups were intraperitoneal injected with curcumol (Shanghai Institute of Material Medical) suspension (dissolved in oil at 5 mg/mL) at 30 mg/kg.^15^ All animals got 24 hours fasting at the end of the experiment and then executed to take blood and liver.

### 2.10 Serum biochemistry

Blood samples were allowed to stand for 1 hours after taken from the eyelids of mice, and serum was extracted after centrifugation and liquated. The serum levels of alkaline phosphatase (ALP), aspartate aminotransferase (AST), alanine aminotransferase (ALT), hyaluronic acid (HA), laminin (LN) and procollagen type III (PCIII) were measured by ELISA kits (Nanjing Yifeixue Biotech).

### 2.11 Histological analysis

Liver tissues were soaked in 5% paraformaldehyde (PFA) and embedded in paraffin. Haematoxylin‐eosin staining (HE) was used for pathological assessments according to the organizational structure. Masson staining and Sirius red staining were used for evaluating collagens. The microscope (ZEISS Axio Vert.A1) was used to take photographs for these staining sections at random fields. Liver tissues were fixed in 0.5% glutaraldehyde and taken photographs at surface of hepatic sinusoid with scanning electron microscope.

### 2.12 Immunofluorescence staining

For cell assays, treated LSECs were fixed with 4% PFA at 37°C for 30 minutes. Then, the cells were permeabilized with 0.1% Triton X‐100 for 5 minutes and blocked with 4% BSA for 30 minutes. Treated cells were co‐incubated with IF‐grade antibody (1:200 dilution) at 4°C overnight and incubated with FITC‐labelled goat anti‐rabbit IgG (1:2000 dilution) or rhodamine‐labelled goat anti‐mouse lgG (1:2000) for 2 hours in the dark. The 4′, 6‐diamidino‐2‐phenylindole (DAPI) was used to stain nucleus. For liver tissues, sections were cut into 5 μm in thickness for further treatment. The CD31 antibody was used to marker LSEC in liver tissues especially. The fluorescence was observed by using fluorescence microscope (Nikon) or confocal microscope (Bio‐Rad Laboratories).

### 2.13 Reagents and antibodies

Cyclopamine and 1‐stearoyl‐2‐arachidonoyl‐snglycerol (SAG) were supplied by Cayman Chemicals; curcumol was supplied by Selleck Chemicals. They were dissolved in DMSO for experiments. The following primary antibodies were used in this study: PDGF‐BB, VEGF‐A, VEGF receptor 2 (VEGFR2), angiopoietin 2, HIF‐1α, CD31, CD34 and PROX1; and SHH, Smo, Gli1 and Hhip1 (Cell Signaling Tech).

### 2.14 Statistical analysis

Data were presented as mean ± SD, and the results were analysed using the GraphPad Prism 7.0 (GraphPad Software). The significance of difference was determined by one‐way analysis of variance with post hoc Dunnett's test. All of the experiments were repeated at least three times on separate occasions. *P* < .05 was considered statistically significant.

## 3 RESULTS

### 3.1 Curcumol inhibits LSEC angiogenesis in vitro

**Figure 1:**
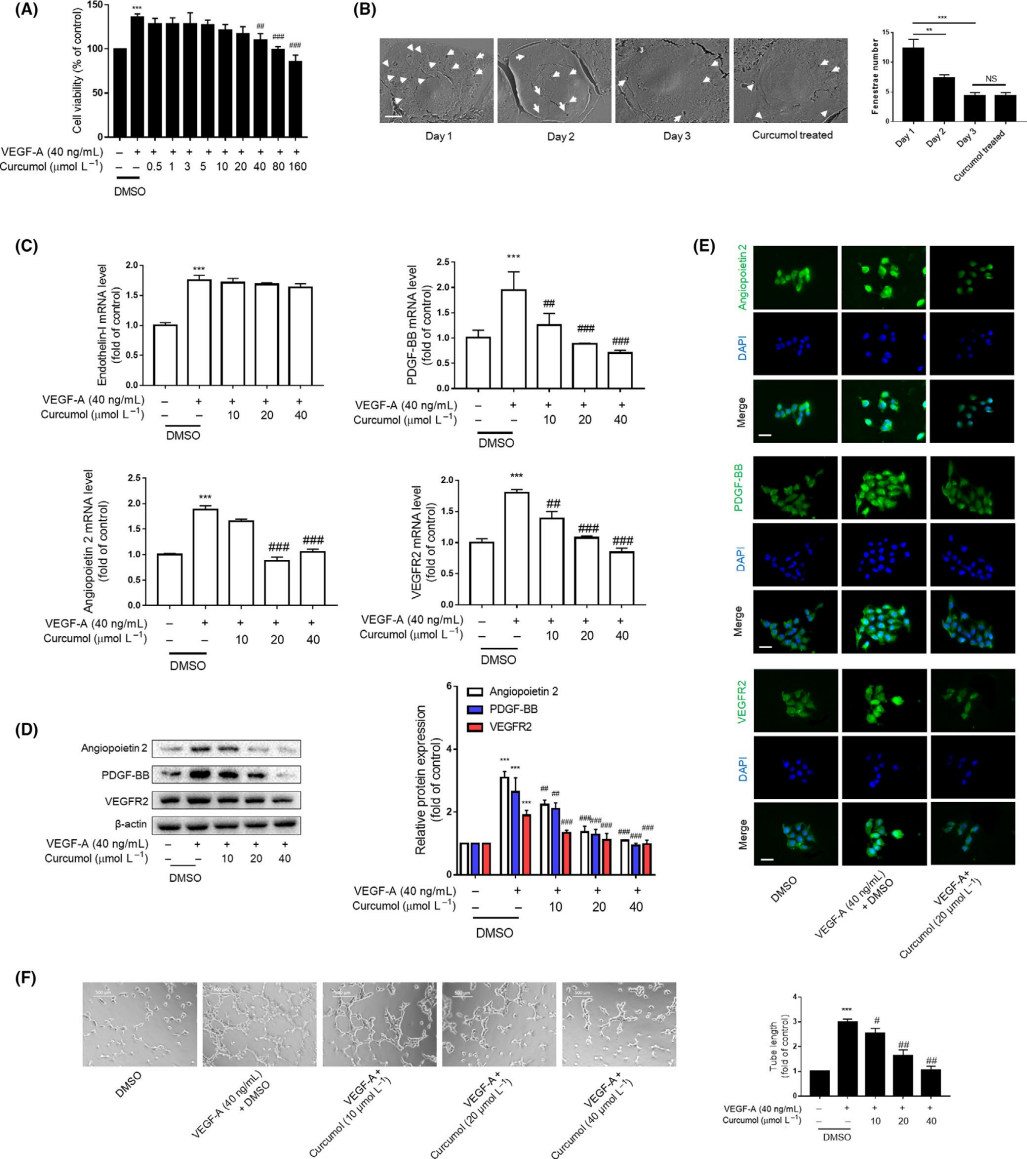
Curcumol inhibits LSEC angiogenesis in vitro. Liver sinusoidal endothelial cells (LSECs) were treated with DMSO or VEGF‐A (40 ng/mL) and different concentrations of curcumol for 24 h. A, Cell viability was determined using MTT. Data were expressed as percentage of control value (n = 6). B, Primary LSECs were cultured on collagen‐covered plates, and the fenestrae were observed with scanning electron microscope scale. Day 1 LSECs show numerous fenestrae (arrowhead). Day 2 and 3 LSECs have few fenestrae. Primary LSECs treated with curcumol (20 μmol/L) for 24 h and cultured for 2 d presented sporadic fenestrae (n = 3). Bar = 5 μm. C, Real‐time PCR analysis of angiogenic cytokines. Data were expressed as fold of control value (n = 4). D, Western blot analyses of angiogenic cytokines (n = 3). E, Immunofluorescence analysis of angiogenic cytokine expression (n = 3). Scale bar = 20 μm. F, Tubulogenesis assay was visualized and quantified with ImageJ (n = 3). Scale bar = 500 μm. ^*^ *P* < .05, ^**^ *P* < .01 and ^***^ *P* < .005 versus control group; ^#^ *P* < .05, ^##^ *P* < .01 and ^###^ *P* < .005 versus model group

In view of the important role of pathological angiogenesis in the occurrence and development of liver fibrosis, the effect of curcumol on LSEC angiogenesis was further investigated. As the hepatic fibrosis occurs, the activated HSCs secrete a large amount of extracellular matrix and the important angiogenic factor VEGF, thereby affecting the perihepatic sinus microenvironment.^12^ More studies revealed that hepatocytes can also express VEGF to change the phenotype of LSEC.^16^ Therefore, we used VEGF‐A (40 ng/mL) in vitro to mimic the living environment of LSECs under pathological condition.^8^ Firstly, cell viability assay provided three rationale dosages of curcumol, at which they did not significantly change cell viability, to be applied to the subsequent experiment in vitro (Figure 1A and Figure S1A). Primary LSECs isolated from rats would lose their fenestrae in 2 days of culture, and medium dose of curcumol (20 μmol/L) had no impact on this progress (Figure 1B). The number and the size of fenestrae of LSECs are based on the oxygen content surroundings. There are various factors controlling LSEC capillarization, and curcumol did not have capacity to reverse this progress in vitro. We found that capillary marker endothelin‐1 mRNA level remained unchanged; however, the angiogenic factor mRNA level was inhibited by curcumol (Figure 1C). Similar results were obtained in terms of the corresponding proteins (Figure 1D,E). Furthermore, the tube formation assay showed that curcumol significantly inhibited LSEC‐mediated tube formation (Figure 1F). Altogether, these data demonstrated that the angiogenic properties of LSECs were negatively regulated by curcumol in vitro.

### 3.2 HIF‐1α is involved in curcumol inhibition of LSEC angiogenesis

**Figure 2:**
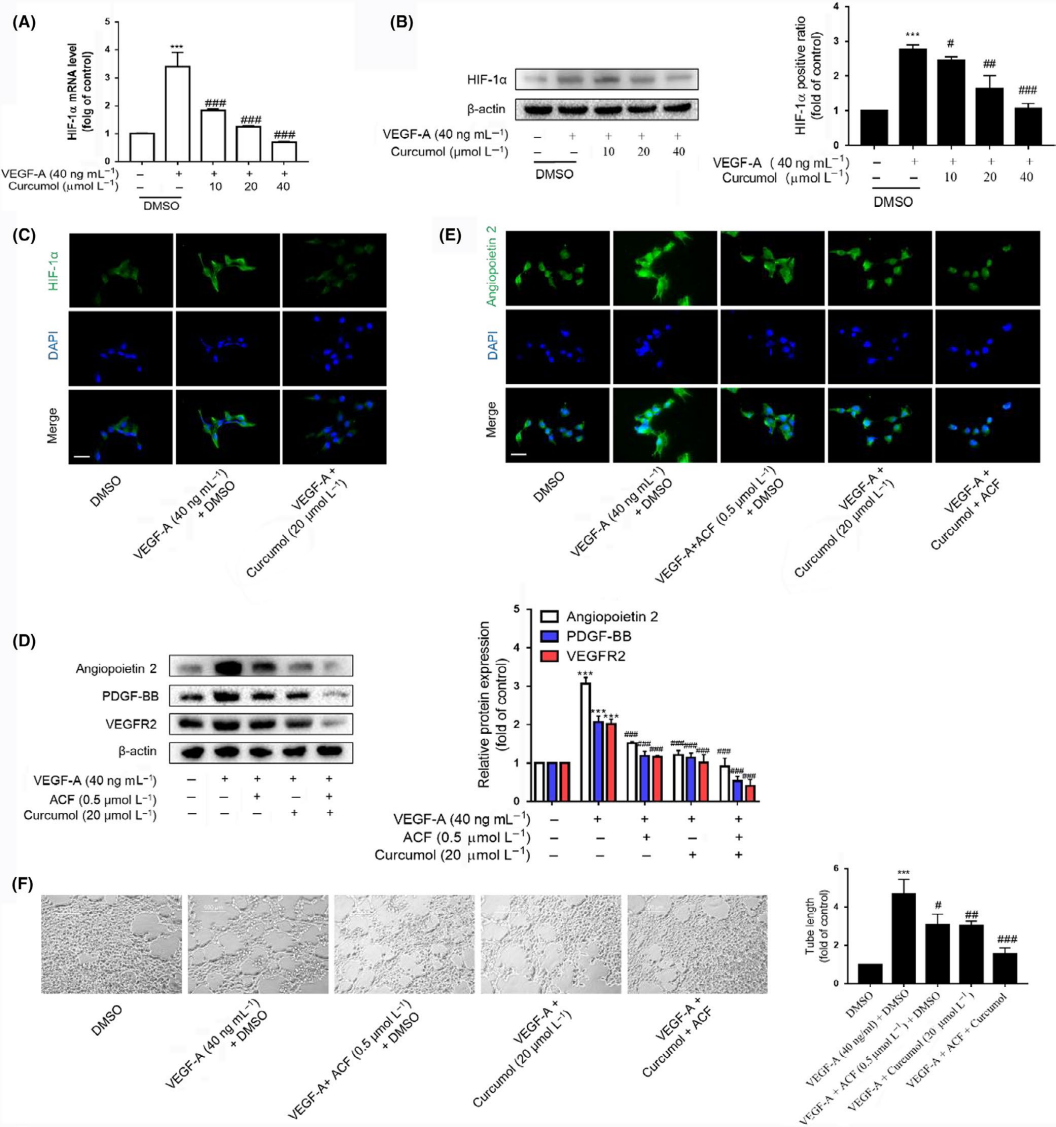
HIF‐1α is involved in curcumol inhibition of liver sinusoidal endothelial cell angiogenesis. A, Real‐time PCR analysis of HIF‐1α. Data were expressed as percentage of control value (n = 4). B, Western blot analyses of HIF‐1α (n = 3). C and E, Immunofluorescence analysis of HIF‐1α and angiogenic cytokines (n = 3). Scale bar = 20 μm. D, Western blot analysis of angiogenic cytokines and ACF (0.5 μmol/L) was utilized to evaluate the function of HIF‐1α (n = 3). F, Tubulogenesis assay was visualized and quantified with ImageJ (n = 3). Scale bar = 500 μm. ^*^ *P* < .05, ^**^ *P* < .01 and ^***^ *P* < .005 versus control group; ^#^ *P* < .05, ^##^ *P* < .01 and ^###^ *P* < .005 versus model group

We next sought to investigate the underlying regulation mechanism of LSEC angiogenesis by curcumol, given that various transcription factors were induced in fibrotic liver due to hypoxia environment.^17^ Previous studies had presented that VEGF was a major target gene of HIF‐1α, and we found hypoxia could increase the expression of HIF‐1α, VEGF‐A and VEGFR2 (Figure S2A). The curcumol decreased the mRNA and protein levels of HIF‐1α after VEGF‐A induction (Figure 2A–C). Interestingly, we found that VEGF‐A failed to change the HIF‐1α level of LSEC living at low density (Figures S2B and S3A). It may be that rapid cell growth induced by VEGF‐A leads to high‐density LSEC hypoxia. Then, we used a HIF‐1α inhibitor named acriflavine (ACF) to confirm the role of HIF‐1α in LSEC angiogenesis. ACF (0.5 μmol/L) reduced the rise of pro‐angiogenic properties of LSECs induced by VEGF‐A that were equal to curcumol (Figure 2D). Furthermore, immunofluorescence staining for angiopoietin 2 had obtained a similar result (Figure 2E). The tube formation assay also showed that ACF (0.5 μmol/L) could decrease the length of the tubes that resemble the effect of curcumol (Figure 2F). Overall, these data convincingly revealed that curcumol inhibited pro‐angiogenic properties of LSECs by regulating HIF‐1α.

### 3.3 Curcumol inhibits LSEC angiogenesis by inhibiting Hh signalling pathway

**Figure 3:**
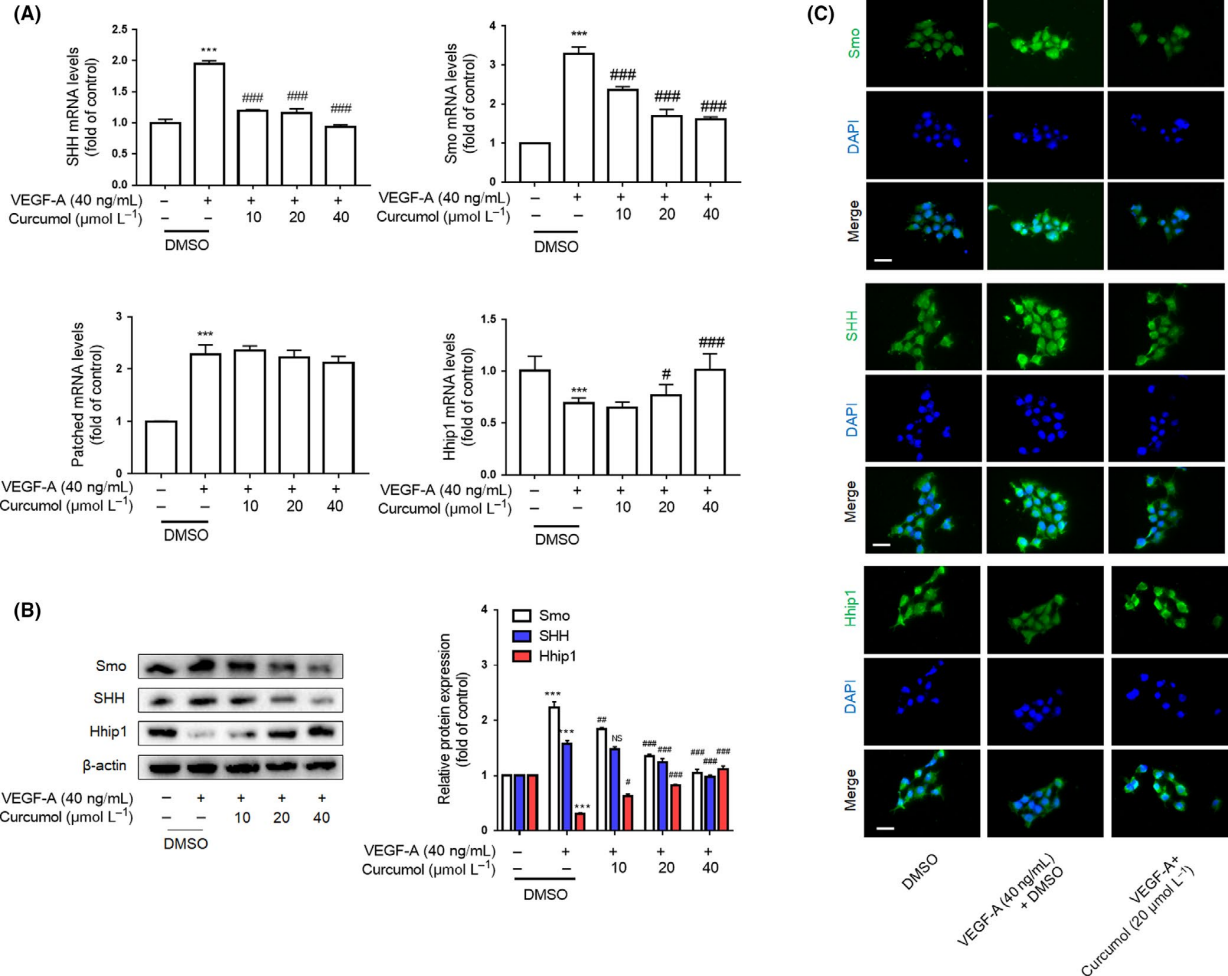
Curcumol inhibits liver sinusoidal endothelial cell angiogenesis by inhibiting Hh signalling pathway. A, Real‐time PCR analysis of hedgehog signalling pathway markers SHH, Smo and Hhip1 (n = 3). B, Western blot analysis of SHH, Smo and Hhip1 (n = 3). C, Immunofluorescence analysis of SHH, Smo and Hhip1 (n = 3). Scale bar = 20 μm. ^*^ *P* < .05, ^**^ *P* < .01 and ^***^ *P* < .005 versus control group; ^#^ *P* < .05, ^##^ *P* < .01 and ^###^ *P* < .005 versus model group

**Figure 4:**
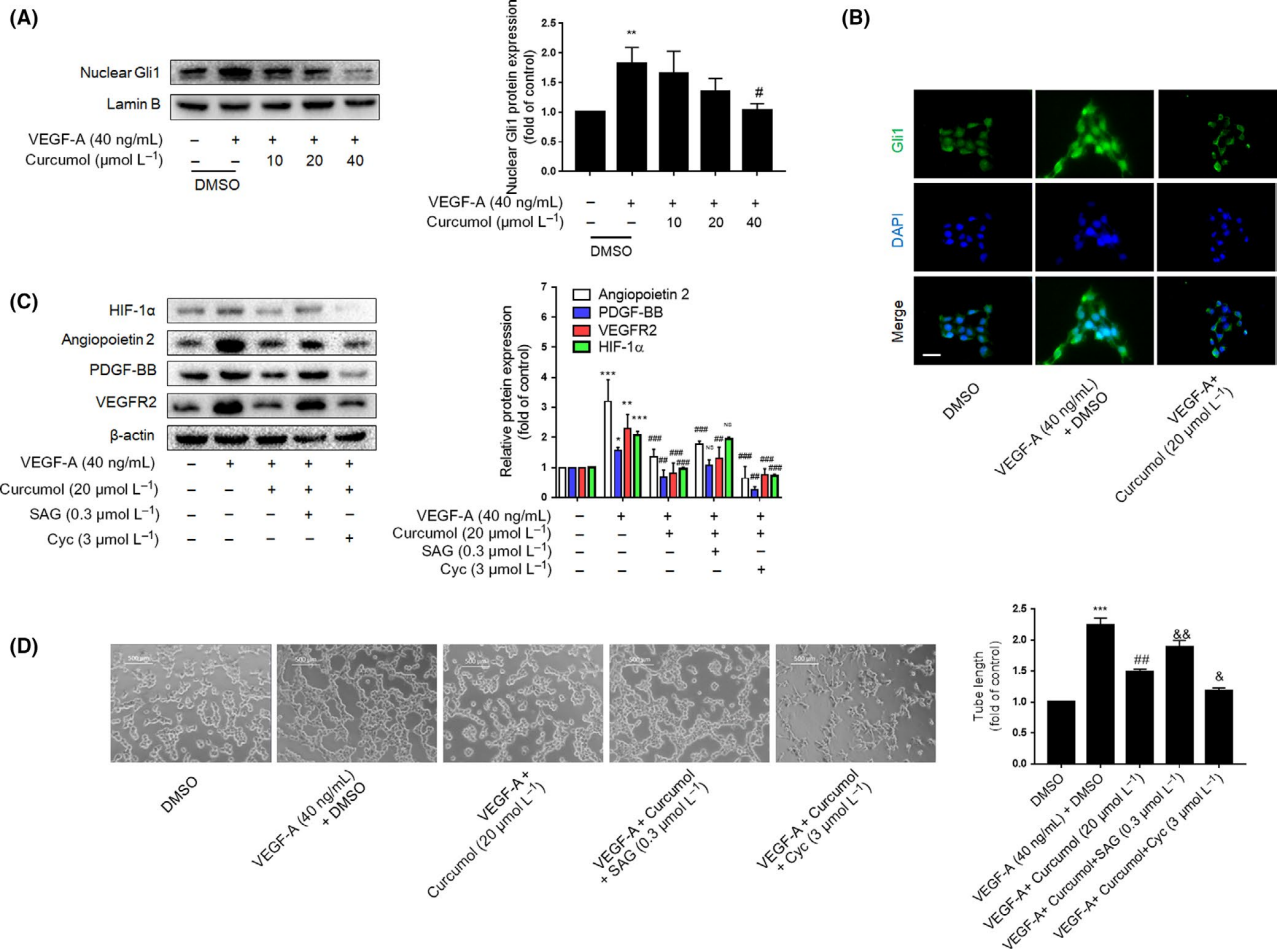
Curcumol inhibits liver sinusoidal endothelial cells (LSECs) angiogenesis by inhibiting Hh signalling pathway. A, Western blot analysis of transcription factor Gli1 expression in nucleus (n = 3). B, Immunofluorescence analysis of Gli1 (n = 3). Scale bar = 20 μm. C, Western blot analysis of HIF‐1α and angiogenic properties of LSECs treated with Hh signalling agonist (SAG 0.3 μmol/L) or inhibitor (cyclopamine 3 μmol/L) (n = 3). D, Tubulogenesis assay was visualized and quantified with ImageJ (n = 3). Scale bar = 500 μm. ^*^ *P* < .05, ^**^ *P* < .01 and ^***^ *P* < .005 versus control group; ^#^ *P* < .05, ^##^ *P* < .01 and ^###^ *P* < .005 versus model group

As we know, activated HIF‐1α transcription and enhanced HIF‐1α protein stability could lead to an increase in HIF‐1α expression.^18^ Different factors could influence HIF‐1α gene transcription. Here, we investigated whether curcumol mediates LSEC angiogenesis via regulating Hh signalling pathway. Our results demonstrated that increased expression of hedgehog signalling markers such as SHH and Smo was attenuated by curcumol dose‐dependently at mRNA and protein levels, whereas the Hhip1, an antagonist protein of hedgehog signalling, experienced opposite result by treatment of curcumol (Figure 3A,B). But we also found that curcumol did not change the level of Patched significantly. Moreover, curcumol could decrease the abundance of Gli1 in nucleus of LSECs, which impaired the hedgehog signalling transcription‐promoting activity (Figure 4A). Immunofluorescence staining assays also confirmed above (Figures 3C and 4B). Subsequently, to further insure whether hedgehog signalling directly participates in the impairment of LSEC angiogenesis by curcumol, we used cyclopamine at 3 μmol/L and SAG at 0.3 μmol/L to block or sensitize hedgehog signalling pathway. As shown in Figure 4C, SAG could offset the inhibiting effect of curcumol on LSEC angiogenesis, whereas cyclopamine exerted synergistic action with curcumol. Nuclear transcription factor HIF‐1α that functions as upstream of angiogenic genes was regulated by hedgehog signalling. As expected, the tube formation assay also confirmed the capacity of hedgehog signalling on LSEC angiogenesis (Figure 4D). Collectively, these findings provided a support that Hh signalling pathway was critically involved in curcumol‐mediated LSEC angiogenesis.

### 3.4 PROX1 regulated by hedgehog signalling pathway maintains accumulation of HIF‐1α in LSECs

**Figure 5:**
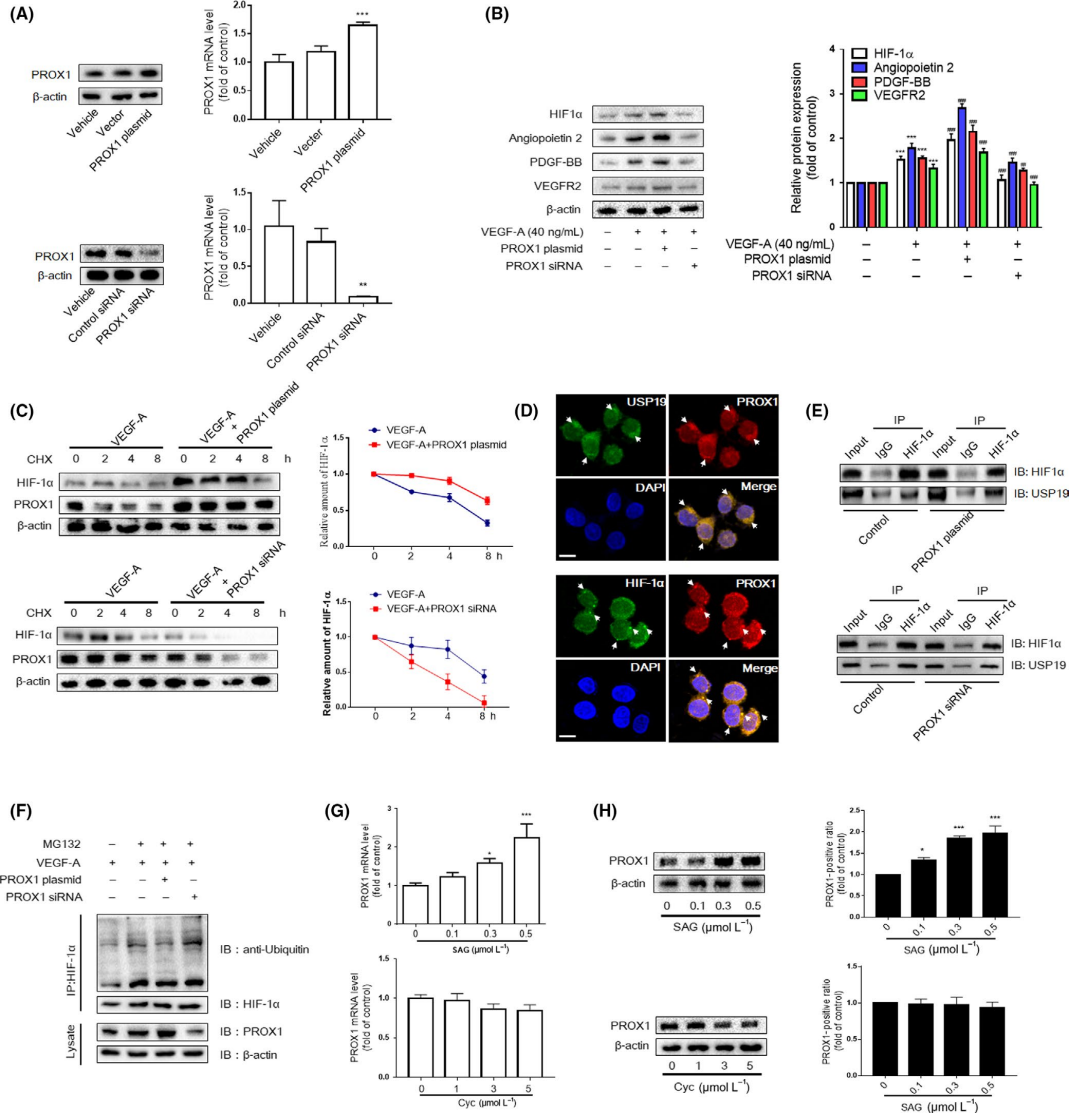
PROX1 regulated by hedgehog signalling pathway maintains accumulation of HIF‐1α in liver sinusoidal endothelial cells (LSECs). A, Western blot and real‐time PCR analyses of efficiency of PROX1 siRNA and overexpression plasmid. B, Western blot analysis of HIF‐1α and angiogenic properties of LSECs treated with PROX1 siRNA and plasmid (n = 3). C, LSECs were infected with the vector plasmid and PROX1 siRNA or overexpression plasmid; then, the CHX (100 mg/mL) was used for the indicated time. Western blot analysis of HIF‐1α and PROX1 (n = 3). Means ± SD from three independent experiments were presented. D, The endogenous PROX1 and HIF‐1α/USP19 were visualized via confocal microscopy in primary LSECs (n = 3). Scale bar = 5 μm. E, Co‐IP assays were carried out with anti‐HIF‐1α and lgG as non‐specific control. The pull‐down lysate was detected using HIF‐1α and USP19 antibody. F, LSECs were treated with protease inhibitor MG132 (10 mg/mL) for 6 h after drug treatment. Co‐IP assays were conducted with anti‐HIF‐1α, followed by detection of the ubiquitin‐conjugated HIF‐1α with ubiquitin antibody. G and H, Western blot and real‐time PCR analyses of PROX1 in LSECs treated with Hh signalling agonist (SAG 0.3 μmol/L) or inhibitor (cyclopamine 3 μmol/L) (n = 3). ^*^ *P* < .05, ^**^ *P* < .01 and ^***^ *P* < .005 versus control group; ^#^ *P* < .05, ^##^ *P* < .01 and ^###^ *P* < .005 versus model group

Numerous studies have identified PROX1 as an important promoter of HCC angiogenesis,^19^, ^20^ and the levels of PROX1 were positively correlated with HIF‐1α protein stability.^21^ Therefore, we speculated that PROX1 could maintain accumulation of HIF‐1α in LSECs and promote LSEC angiogenesis. Firstly, transfection efficiency of PROX1 siRNA or PROX1 plasmid was analysed by determining the levels of protein and mRNA, and the results demonstrated that the PROX1 successfully decreased or increased in LSECs, respectively (Figure 5A). Transfecting LSECs after treated with VEGF‐A, we found that PROX1 siRNA apparently repressed the expression of HIF‐1α and pro‐angiogenic factors in LSECs, whereas PROX1 plasmid elevated their expression (Figure 5B).

The PROX1‐mediated elevation in HIF‐1α level might be due to an enhanced stability of HIF‐1α protein. To assess this possibility, we determined the level of HIF‐1α at different time points after LSECs were treated with the translation inhibitor cycloheximide (CHX). The half‐life of HIF‐1α was substantially prolonged in PROX1‐overexpressing LSECs, while it was significantly shortened in PROX1‐knockdown LSECs (Figure 5C). Since HIF‐1α is degraded through an ubiquitin‐dependent mechanism, we wondered that PROX1 can inhibit HIF‐1α ubiquitin by recruiting a deubiquitinase called USP19. Interestingly, PROX1 was found to co‐localize with USP19 and/or HIF‐1α in LSECs via confocal laser scanning microscope (Figure 5D). Moreover, the amount of USP19 co‐precipitated with HIF‐1α was increased in PROX1‐overexpressing LSECs, while it was diminished in PROX1‐knockdown LSECs (Figure 5E). These results suggested that PROX1 inhibits HIF‐1α ubiquitination via its association with USP19. We further examined the level of HIF‐1α ubiquitination. Ubiquitin‐conjugated HIF‐1α was pulled down by anti‐HIF‐1α and subjected to Western blotting with anti‐ubiquitin. Upon PROX1 overexpression, the quantity of the ubiquitin‐conjugated HIF‐1α was markedly diminished, and an opposite experimental result was found in PROX1‐knockdown LSECs (Figure 5F). In addition, we found that the agonist of hedgehog signalling increased the mRNA and protein levels of PROX1 significantly; the antagonist, however, had a feeble effect (Figure 5G,H). We concluded that the primary LSECs express little activity of hedgehog signalling so that they react to its antagonist inactively. Taken together, these results indicated that transactivation of PROX1 by canonical hedgehog signalling could inhibit HIF‐1α ubiquitination.

### 3.5 Curcumol inhibits LSEC angiogenesis via regulating PROX1

**Figure 6:**
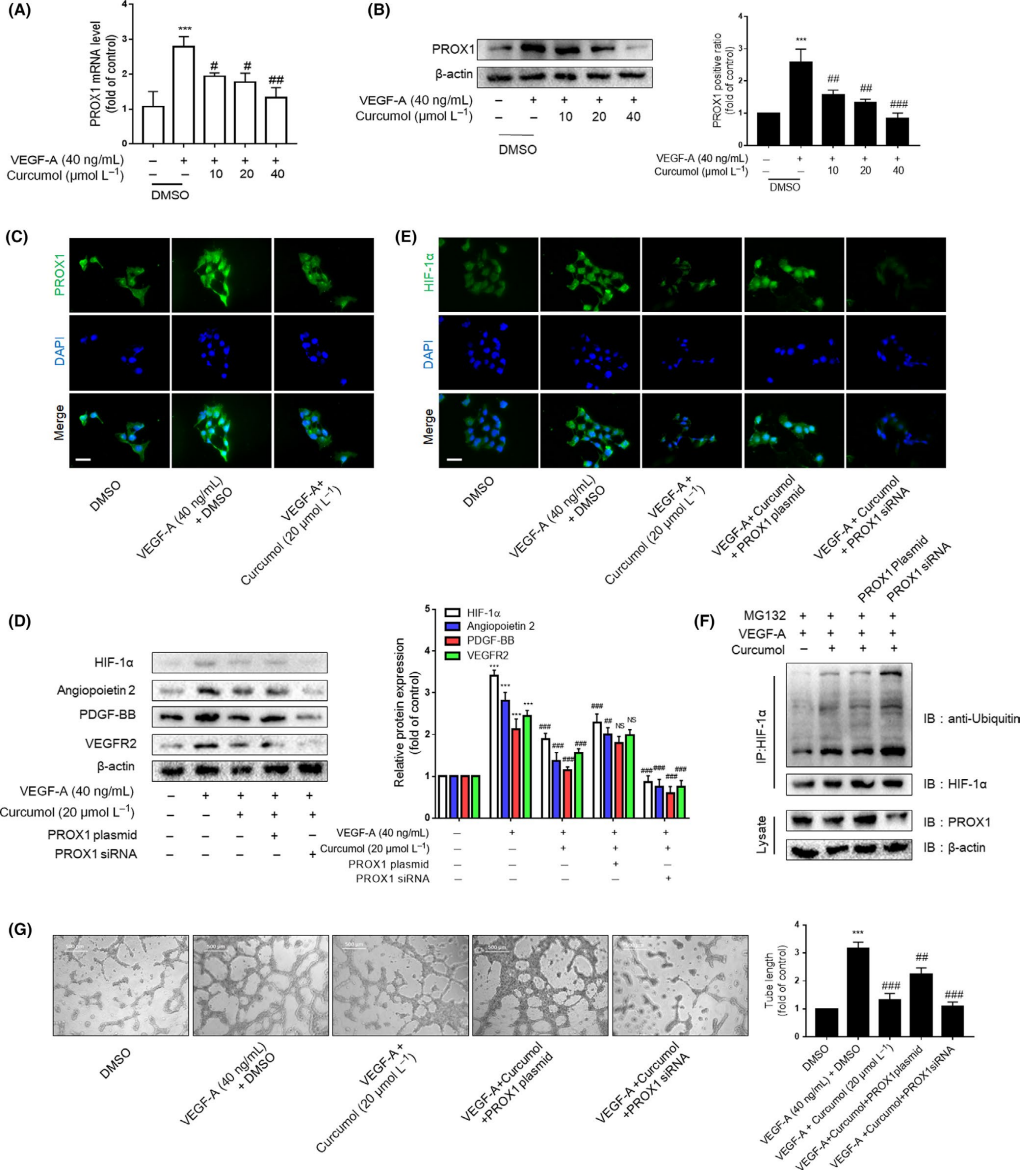
Curcumol inhibits liver sinusoidal endothelial cells (LSECs) angiogenesis via regulating PROX1. A, Real‐time PCR analysis of PROX1 mRNA in DMSO‐ and curcumol‐treated LSECs after the induction of VEGF‐A (n = 3). B, Western blot analysis of PROX1 protein expression in LSECs (n = 3). C and E, Immunofluorescence analysis of PROX1 and HIF‐1α protein expression in LSECs (n = 3). Scale bar = 20 μm. D, Western blot analysis of HIF‐1α and angiogenic properties of LSECs treated with the above models plus PROX1 plasmid (n = 3). F, LSECs were treated with protease inhibitor MG132 (10 mg/mL) for 6 h after drug treatment. Co‐IP assays were conducted with anti‐HIF‐1α, followed by detection of ubiquitin, HIF‐1α and PROX1. G, Tubulogenesis assay was visualized and quantified with ImageJ (n = 3). Scale bar = 500 μm. ^*^ *P* < .05, ^**^ *P* < .01 and ^***^ *P* < .005 versus control group; ^#^ *P* < .05, ^##^ *P* < .01 and ^###^ *P* < .005 versus model group

We next determined the functional contribution of PROX1 to inhibition of LSEC angiogenesis by curcumol. LSECs were treated by similar method as above, and the activation of PROX1 was determined via different technical methods. As shown in Figure 6A–C, PROX1 can be activated by VEGF‐A treatment and inhibited by multiple doses of curcumol significantly. HIF‐1α and its downstream angiogenic factors impaired by curcumol in VEGF‐A–induced LSECs were repaired under PROX1 overexpression, whereas they were further decreased owing to PROX1 downexpression (Figure 6D,E). Moreover, ubiquitination assay also identified that curcumol can strengthen HIF‐1α ubiquitination dependent on PROX1 expression (Figure 6F). The result of the tube formation assay confirmed the effects of PROX1 on LSEC angiogenesis (Figure 6G). Pharmaceutical treatment can increase the protein degradation of HIF‐1α, while PROX1 overexpression abrogates the effect of curcumol. These results showed that LSEC angiogenesis was regulated by curcumol through controlling the level of PROX1.

### 3.6 Curcumol inhibits LSEC angiogenesis and attenuates liver fibrosis in mice

**Figure 7:**
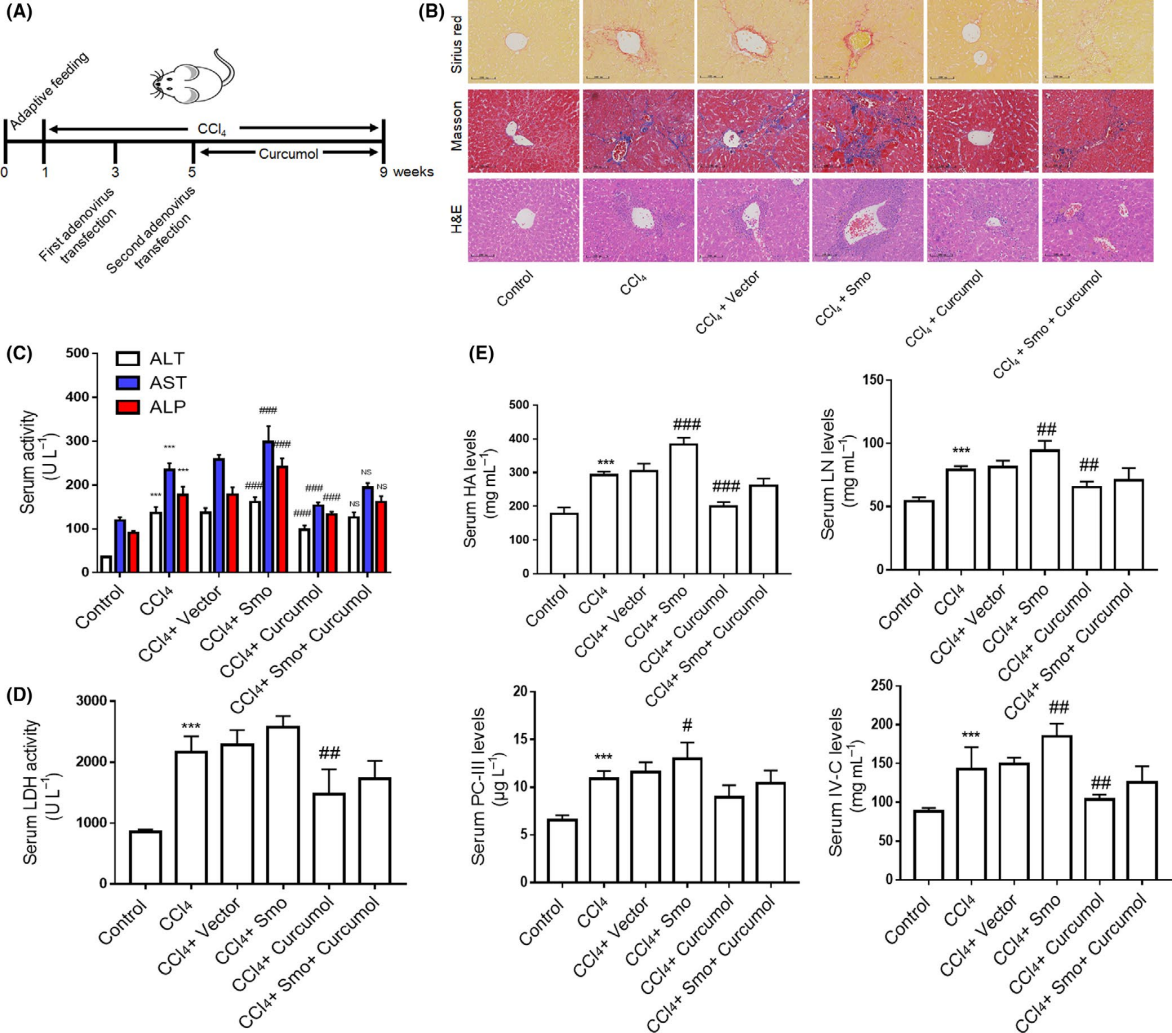
Curcumol attenuates liver fibrosis in mice. A, After a week of adaptive feeding, mice were randomly assigned to six groups and injected i.p. with CCl_4_ except for control group for 8 wk to induce liver fibrosis. The transfection adenoviruses were given twice in the 3rd and 5th weeks, respectively. Besides, 30 mg/kg curcumol was injected i.p. in other day during the last 4 wk. B, Liver sections were stained with Sirius red reagents, Masson reagents and H&E for collagen and histological examinations (n = 3). Scale bar = 100 μm. C, Determination of serum levels of liver injury markers ALP, AST and ALT (n = 6). D, Determination of serum levels of hydroxyproline content (n = 6). E, Determination of serum levels of fibrotic markers HA, LN, PC‐Ⅲ and Ⅳ‐C (n = 6). ^*^ *P* < .05, ^**^ *P* < .01 and ^***^ *P* < .005 versus control group; ^#^ *P* < .05, ^##^ *P* < .01 and ^###^ *P* < .005 versus model group

**Figure 8:**
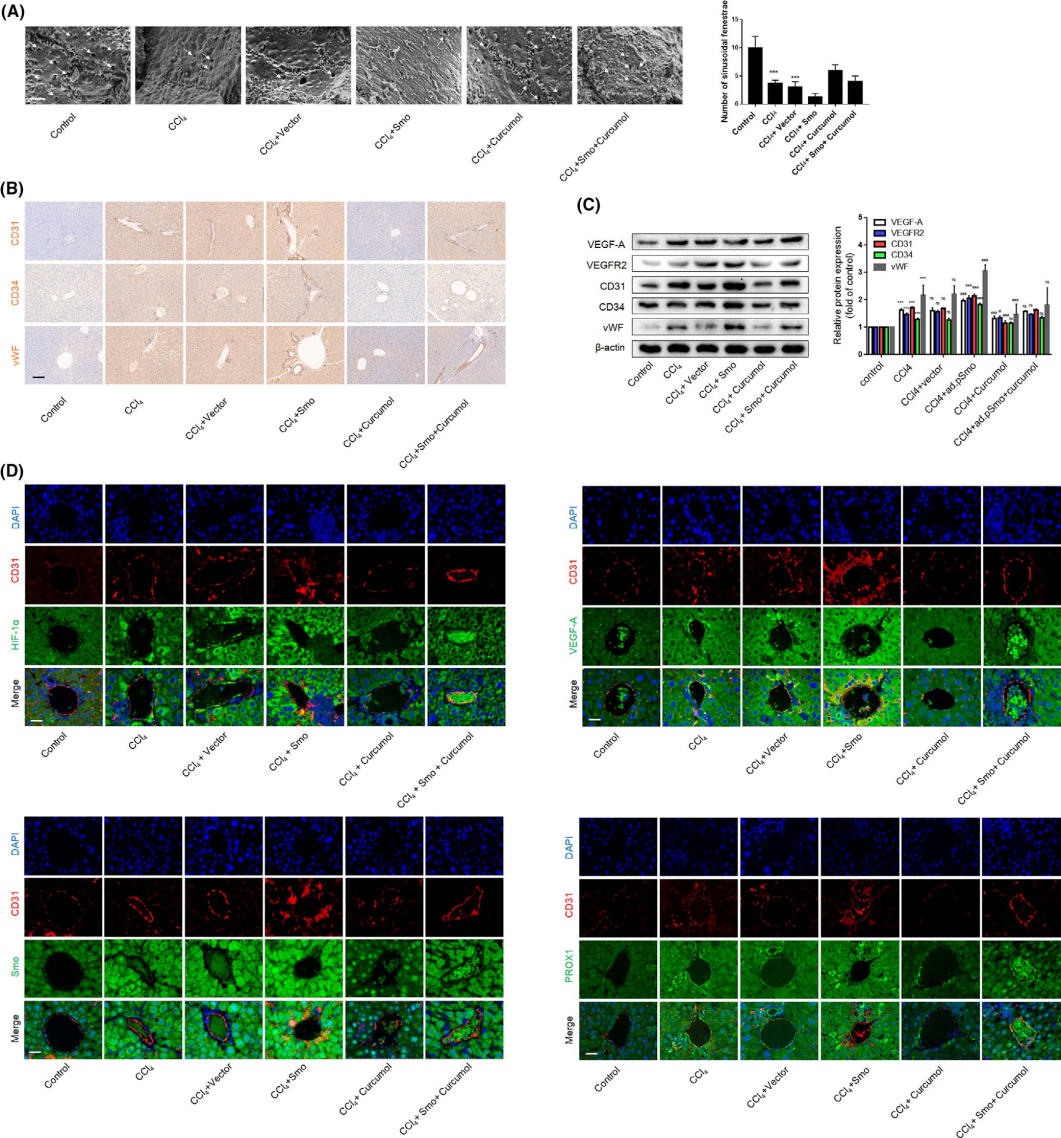
Curcumol inhibits liver sinusoidal endothelial cell angiogenesis through regulating Hh signalling pathway in mice. A, Analysis of sinusoidal fenestrae (arrowhead) in liver tissues (n = 3). Scale bar = 1 μm. B, Immunohistochemical analyses of endothelial markers CD31, CD34 and vWF in liver tissues (n = 3). Scale bar = 100 μm. C, Western blot analysis of endothelial markers and angiogenic factors (n = 3). D, Immunofluorescence analysis of endothelial markers CD31 accompanied by HIF‐1α, VEGF‐A, Smo and PROX1 in liver tissues (n = 3). Scale bar = 20 μm. ^*^ *P* < .05, ^**^ *P* < .01 and ^***^ *P* < .005 versus control group; ^#^ *P* < .05, ^##^ *P* < .01 and ^###^ *P* < .005 versus model group

We finally examined the effect of curcumol in vivo using male mice with CCl_4_‐induced liver fibrosis. Besides, we raised the Smo expression through adenovirus transfection in order to explore the function of hedgehog signalling pathway in hepatic pathological angiogenesis (Figure 7A). Different histological analyses were applied to evaluate liver fibrosis. Sirius red staining, Masson and H&E showed that CCl_4_‐induced liver represented heavier collagen deposition and structural disorder accompanied by an inflammatory response. The mice treated with Smo adenovirus plasmid represented more severe liver injury compared with CCl_4_ alone, while curcumol had ability to alleviate the liver fibrosis (Figure 7B and (Figure S4A,B). These results were confirmed by measuring the serum levels of ALP, AST and ALT (Figure 7C). Exanimations of the serum levels of key fibrogenic indicators HA, LN, PCⅢ and Ⅳ‐C (Figure 7E) and liver hydroxyproline contents (Figure 7D) also presented the same result. LSECs of fibrotic liver or Smo overexpression liver undergone capillarization and lost their fenestrae, while curcumol‐treated mice represented a better recovery (Figure 8A). Strangely, the in vivo result of curcumol regulating LSEC capillarization was different from the in vitro result (Figure 1B). Here, we speculated that curcumol can alleviate the liver fibrosis and improve the liver microenvironment, by which LSECs could maintain their normal phenotype. The protein expressions of CD31, CD34 and vWF, three key endothelial markers indicating angiogenesis, and angiogenic properties such as VEGF‐A and VEGFR2 were increased by CCl_4_ associated with Smo adenovirus plasmid and decreased by curcumol (Figure 8B,C). Similar results were obtained via immunofluorescence staining in which CD31 was utilized to target the LSECs. Activation of Hh signalling could exacerbate mouse liver fibrosis (Figure S5A) and pathological angiogenesis via increasing the levels of HIF‐1α and VEGF‐A; meanwhile, it has a positive effect on PROX1 expression (Figure 8D). In summary, these data provided that curcumol had mitigated hepatic fibrosis and inhibited pathological angiogenesis, and the hedgehog signalling pathway and PROX1 are involved in this progress.

## 4 DISCUSSION

Liver fibrosis usually occurs in chronic liver diseases and is a vital driver of liver cirrhosis and hepatocellular carcinoma. As significant progress has been made in confirming and intervening the aetiologies of liver fibrosis, more researchers draw attention to HSCs and a variety of therapies exist to inhibit or reverse phenotype of activated HSCs.^22^ Vascular endothelium represents the interface between blood and other tissues of the body. Not only it is a physical barrier but also it is implicated in different physiological roles,^23^ such as haemostasis/ thrombosis,^24^ metabolites transportation, inflammation,^25^ angiogenesis and vascular tone.^26^, ^27^ Liver endothelium is made up of LSECs and adjacent HSCs. Phenotypic stability of LSECs is vital to maintain quiescent HSCs and protect hepatocytes.^16^, ^28^ Hepatic sinusoidal angiogenesis owing to dysfunctional LSECs accompanied by an abnormal angioarchitecture is a symbol related to liver fibrogenesis, which indicates a potential target for therapeutic interventions.^3^ For instance, sorafenib,^29^ a tyrosine‐kinase inhibitor, has been proved to block sinusoid remodelling and facilitate liver fibrosis resolution. Recent studies also explained that different active components in natural medicinal plants could alleviate liver fibrosis by inhibiting pathological angiogenesis.^30^, ^31^, ^32^, ^33^ We previously reported that curcumol mitigated liver pathological changes via promoting activated HSC necroptosis.^15^ However, the effects of curcumol on LSEC angiogenesis in liver fibrosis had not been investigated, and the underlying mechanism remained to be uncovered. In this article, we confirmed firstly that curcumol ameliorated angiogenesis of LSECs in a murine liver fibrosis model.

As we know, a large amount of nutrients and oxygen are consumed in the process of angiogenesis.^34^ To adapt to hypoxia, cells reduce oxygen consumption and maintain homeostasis that are mediated by HIF‐1α.^17^ We investigated a potential mechanism of curcumol anti‐angiogenesis effect and identified HIF‐1α as a key upstream regulator of angiogenic properties. In view of the results, the primary LSECs treated with angiogenesis stimulator VEGF‐A possessed of higher protein expression and mRNA level of HIF‐1α, while curcumol regulated the HIF‐1α reversely. Moreover, ACF, a HIF‐1α inhibitor, was used to confirm the effect of HIF‐1α. Here, we have demonstrated that HIF‐1α is an upstream mediator of angiogenic properties and serves as an essential factor of the curcumol‐regulated anti‐angiogenic network.

The activation of HIF‐1α transcription and the stabilization of HIF‐1α protein are essential for HIF‐1α translocating to nuclear and regulating the activation of target genes. As we reported previously, Glis family transcription factors of canonical Hh pathway could enter nuclear and activate target genes including HIF‐1α in HSCs.^31^ Here, we found that curcumol can inhibit the markers of Hh pathway dose‐dependently in LSECs, and agonist or antagonist of this signalling pathway was used to confirm the effect of the canonical Hh pathway on HIF‐1α protein expression. Curcumol functioned as an inhibitor of HIF‐1α through declining the nuclear level of Gli1. Besides, non‐canonical Hh pathway appeared to contribute to cholangiocarcinoma progression, which implied us that Hh has a great influence on the microenvironment of liver.^35^ HIF‐1α is kept under tight regulation, and in normal physiological condition, it is the most short‐lived proteins known. The ubiquitin‐proteasome system practically kept steady‐state levels low. Until recently, studies of post‐translational modifications of HIF‐1α were expanded to hydroxylation,^36^ phosphorylation,^37^ methylation 38 and acetylation.^39^ In hepatocellular carcinoma, the homeobox protein PROX1 is overexpressed in hepatocytes and is required for cell migration. The results of assays suggested that the amino‐terminal two‐thirds (amino acids 1‐570) of PROX1 was responsible for the interaction with HIF‐1α, upregulating HIF‐1α expression to induce EMT response in HCC cells.^21^ Another research had showed that PROX1 could prevent p65 ubiquitination by recruiting USP7 to inhibit HCC angiogenesis.^19^ Therefore, we focused on the ubiquitination of HIF‐1α that a reversible process is dependent on deubiquitylating enzymes (DUBs). As regards USP19 regulating HIF‐1α during hypoxia,^40^ we investigated the relationship between PROX1 and USP19. According to the result, we had demonstrated that PROX1 could inhibit ubiquitination of HIF‐1α by recruiting USP19, and firstly reported that curcumol could downregulate PROX1 expression to maintain accumulation of HIF‐1α. Further researches showed that activation of hedgehog signalling by SAG resulted in increased expression of PROX1, and Smo overexpression in murine increased the protein level of PROX1 in vivo. These data suggested that curcumol restrains LSEC‐mediated angiogenesis through inhibiting hedgehog signalling pathway associated with decreasing PROX1 protein expression.

It is noteworthy that many controversial issues still remained unsolved among this research. We confirmed the anti‐angiogenic effect of curcumol through Hh signalling pathway; however, the direct target of drug was still unknown. Glis family transcription factors could affect various genes in nuclear, but the specific regulation mechanism had not been revealed. The effect of curcumol on the LSEC capillarization in vitro and in vivo is inconsistent. The maintenance of fenestrae structure depends on various factors. The loss of fenestrae in the cultured LSECs was rescued by silencing DLL4 in vitro,^41^ and BMP9 is a key paracrine secreted from HSCs to control LSEC fenestration.^42^ Primary LSECs almost pursued to lose their fenestrae out of normal environment regardless of the slight effect of curcumol in vitro. The process of hepatic angiogenesis is divided into many stages, and the effects of drug therapy on angiogenesis at different stages are also different.^7^ LSEC renewal differs in physiological and in pathological conditions.^43^ It is impressively difficult to distinguish the characteristics of angiogenesis between liver regeneration and liver cirrhosis. According to our experiment in vivo, curcumol was given to mice in the early stage of liver fibrosis and showed great resistance to hepatic fibrosis and angiogenesis. However, we still think that curcumol could have multiple targets to achieve its pharmaceutical characteristics. Our previous research had investigated whether curcumol is able to alleviate liver fibrosis via triggering HSC necroptosis.^15^ Activated HSCs can secrete angiogenic factors to regulate LSECs, and it also participates in liver angiogenesis directly.^12^ Inhibition of activated HSCs by curcumol would attenuate LSEC‐mediated angiogenesis. LSECs account for the majority of liver non‐parenchymal cells and are the most important part of neovascularization, so we draw a conclusion that curcumol could alleviate liver fibrosis via inhibiting LSEC‐mediated angiogenesis. A future perspective could be represented by the investigation of curcumol for the modulation of cellular processes involved in LSEC‐mediated angiogenesis in the liver.

**Figure 9:**
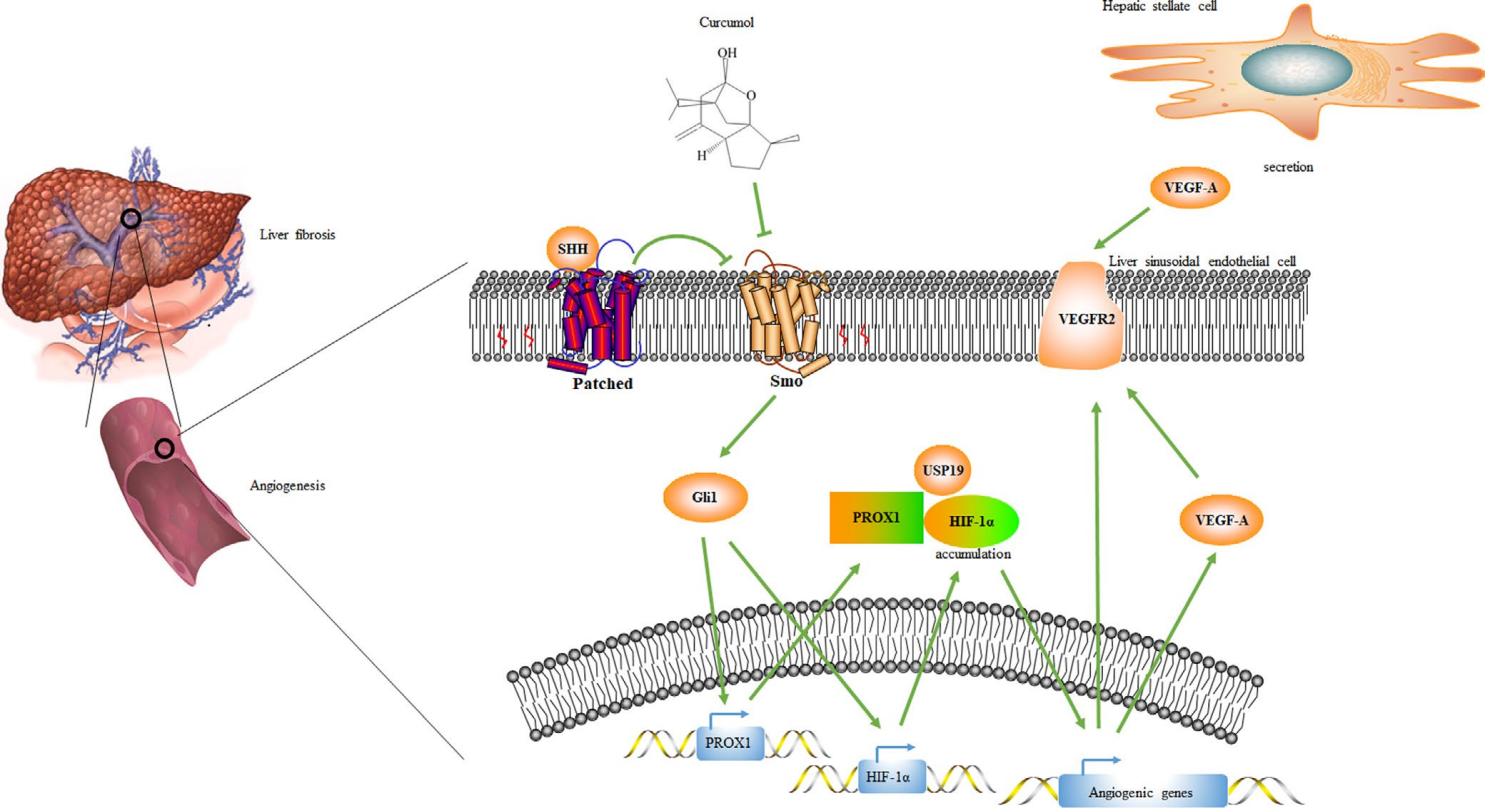
Diagram illustrates the mechanisms of curcumol regulating liver sinusoidal endothelial cell‐mediated angiogenesis in liver fibrosis

In summary, we demonstrated that canonical hedgehog signalling regulated LSEC angiogenesis through transactivation of PROX1 and HIF‐1α. Natural medicine curcumol could alleviate sinusoid pathological angiogenesis by impairing hedgehog signalling (Figure 9). These findings provided a novel molecular mechanism for curcumol to be a new anti‐fibrotic agent for liver fibrosis.

## CONFLICT OF INTERESTS

All authors confirm that there is no conflict of interest.

## AUTHOR CONTRIBUTIONS

SZ, FZ and XY designed the research. XY, ZW and JK made the constructs and performed assays. XS participated in animal experiments. YJ and FW provided fundamental research of curcumol. JS, ST and AC made computational calculation associated with collecting the data. XY wrote the manuscript. ZZ and SW revised the manuscript. All authors read and approved the final manuscript.

## Data Availability

All data generated or analysed during this study are included in this article.
