# Supplementary material for: Curcumol attenuates liver sinusoidal endothelial cell angiogenesis via regulating Glis‐PROX1‐HIF‐1α in liver fibrosis
Source: Cell Prolif. 2020 Mar 2;53(3):e12762. doi: 10.1111/cpr.12762 (PMC7106966; doi:10.1111/cpr.12762)
Supplement: Supplementary file 1 [file CPR-53-e12762-s001.docx]

**
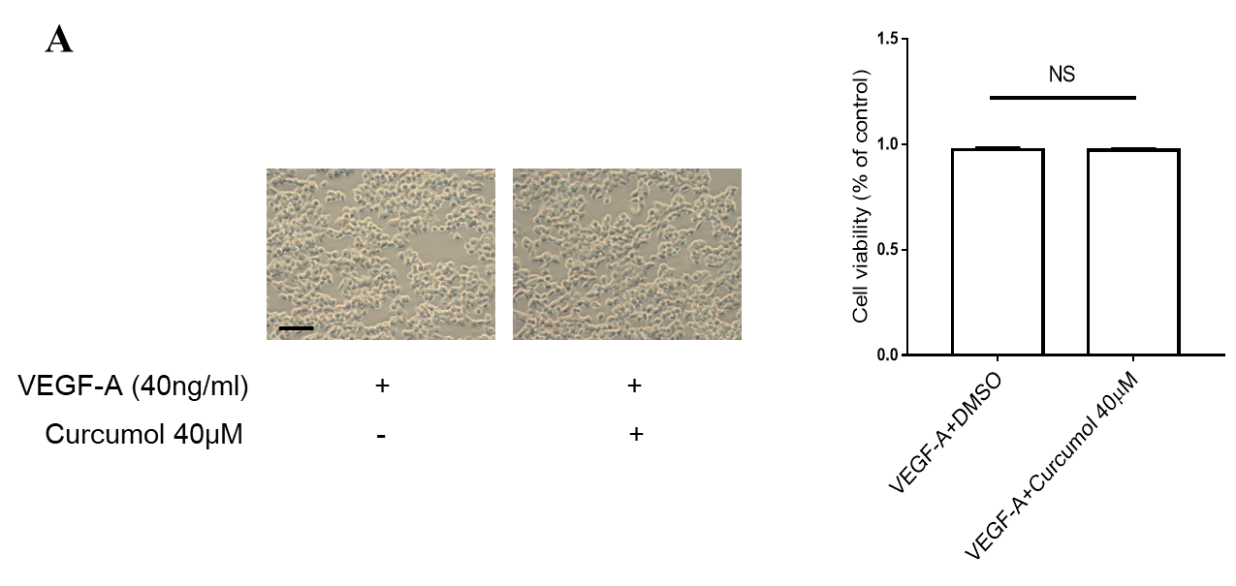
**

**Supplementary Figure 1** (A) After curcumol treatment for 24h, LSECs were digested using trypsin without EDTA, and gathered by centrifugation, then washed twice with PBS and suspended in PBS. Cell suspension and trypan blue solution were mixed gently in 9:1 ratio and stained for 2 min. Scale bar, 100 μM. Data are expressed as mean ± SD (n=3).


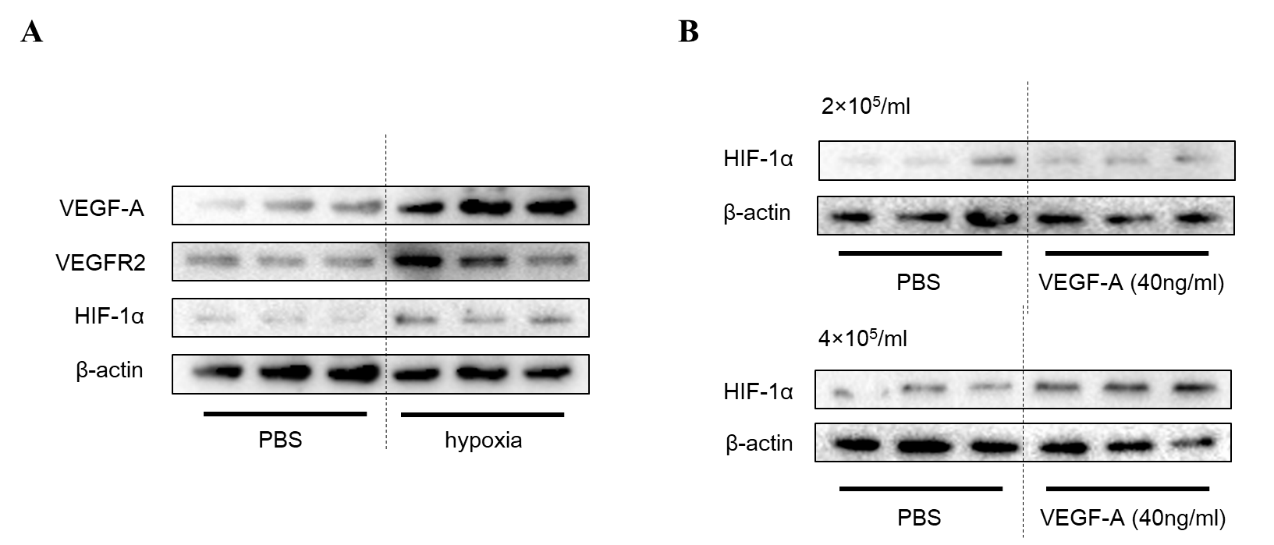


**Supplementary Figure 2** (A) LSECs were cultured in 5% O_2_ and 5 % CO_2_ incubator for 12 hours. The protein solution were extracted rapidly for western blot assay (n=3). (B) Added 40ng/ml VEGF-A into culture medium of LSECs at different density, protein expression of HIF-1α was confirmed via western blot assay (n=3).


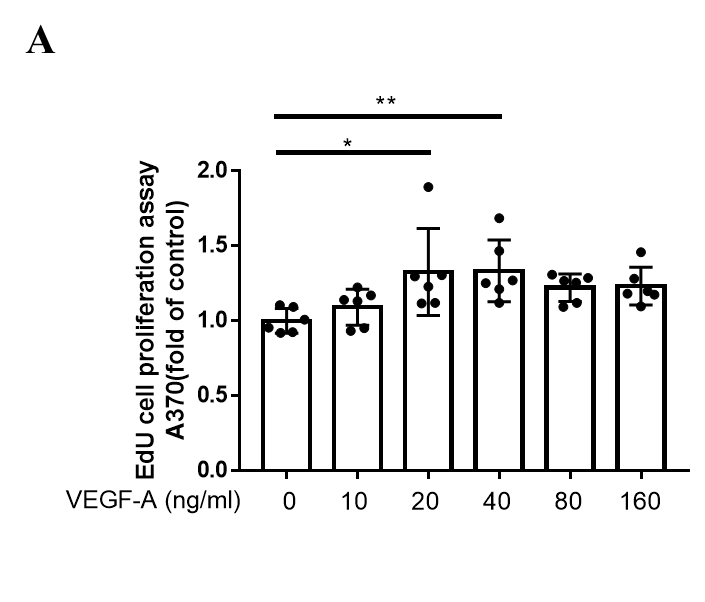


**Supplementary Figure 3** (A) LSECs were cultured up to 50% growth density in 96 wells plate and treated with VEGF-A at different concentrations for 24 h. The cell proliferation ability of treated LSECs were measured with EdU cell proliferation kit. Detected the absorbance at 370nm (n=6). *P<0.05, **P<0.01 versus control group.


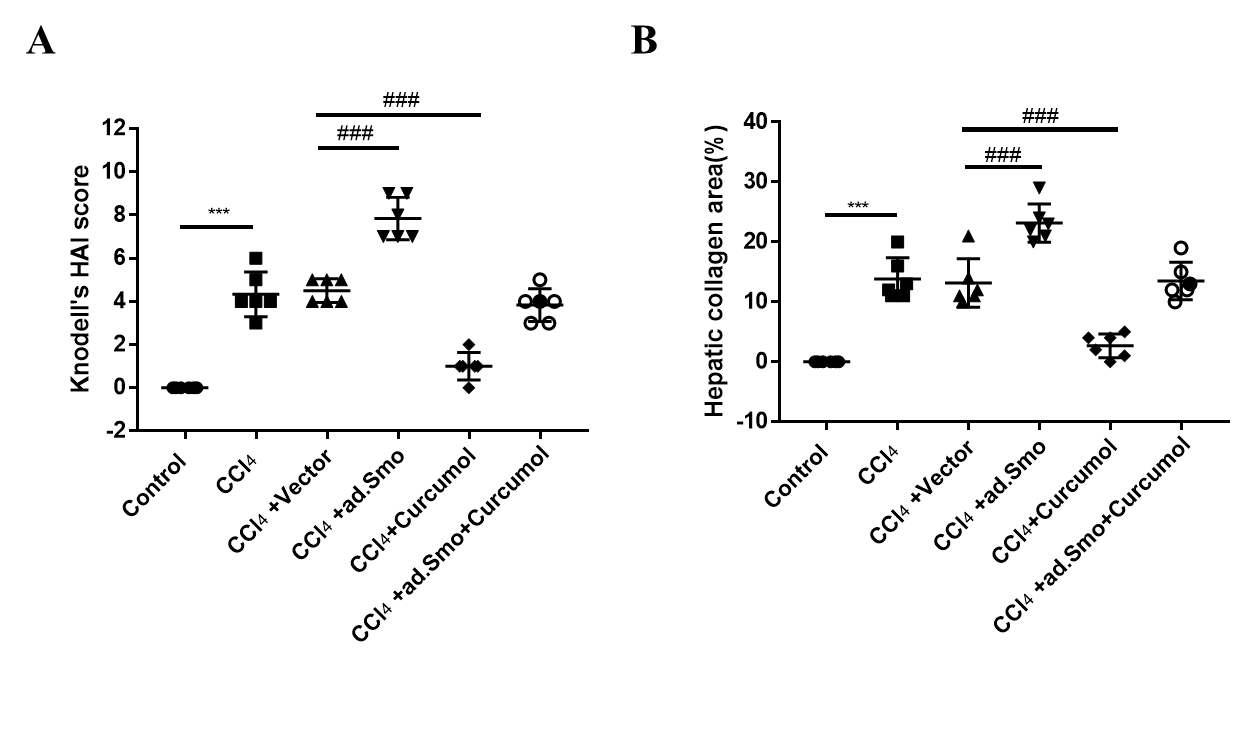


**Supplementary Figure 4** (A) Ishak fibrosis scores, knodell’s histological activity index according to the HE results (n=6). (B) Collagen area had been determined based on Masson trichrome staining and quantified with Image J (n=6). ***P<0.005 versus control group; ###P<0.005 versus CCl_4_ + Vector group.


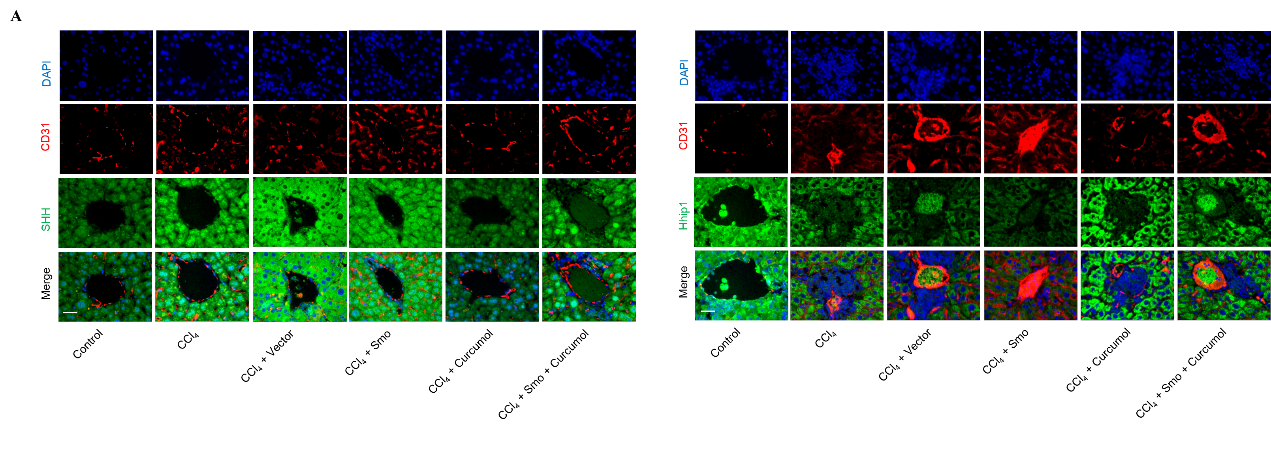


**Supplementary Figure 5** (A) Immunofluorescence analysis of endothelial markers CD31 companied by SHH and Hhip1 in liver tissues (n=3). Scale bar=20μm.
